# Community lung health service design for COPD patients in China by the Breathe Well group

**DOI:** 10.1038/s41533-022-00286-8

**Published:** 2022-08-19

**Authors:** Hui Pang, Zihan Pan, Rachel Adams, Eleanor Duncan, Chunhua Chi, Xia Kong, Peymané Adab, Kar Keung Cheng, Brendan G. Cooper, Jaime Correia-de-Sousa, Andrew P. Dickens, Alexandra Enocson, Amanda Farley, Nicola Gale, Kate Jolly, Sue Jowett, Mariam Maglakelidze, Tamaz Maghlakelidze, Sonia Martins, Alice Sitch, Katarina Stavrik, Raphael Stelmach, Alice Turner, Siân Williams, Rachel E. Jordan

**Affiliations:** 1 Department of General Practice, Peking University First Hospital, 100034 Beijing, China; 2 Department of Emergency, Beijing Friendship Hospital, Capital Medical University, 100050 Beijing, China; 3 Pulmonary and Critical Care Medicine, Peking University Third Hospital, 100191 Beijing, China; 4 Research Center for Chronic Airway Diseases, Peking University Health Science Center, Beijing, China; 5 Institute of Applied Health Research, University of Birmingham, B15 2TT Birmingham, UK; 6 Lung Function & Sleep, Queen Elizabeth Hospital, B15 2WB Birmingham, UK; 7 International Primary Care Respiratory Group, Aberdeenshire, UK; 8 Life and Health Sciences Research Institute (ICVS), School of Medicine, University of Minho, Braga, Portugal. ICVS/3B’s, PT Government Associate Laboratory, Braga/Guimarães, Portugal; 9 Georgian Respiratory Association, Tbilisi, Georgia; 10 Petre Shotadze Tbilisi Medical Academy, Tbilisi, Georgia; 11 Ivane Javakhisvhili Tbilisi State University, Tbilisi, Georgia; 12 Family Medicine, ABC Medical School, Sao Paulo, Brazil; 13 NIHR Birmingham Biomedical Research Centre, University Hospitals Birmingham NHS Foundation Trust and University of Birmingham, Birmingham, UK; 14 Center for Family Medicine, Faculty of Medicine, Ss.Cyril and Methodius University in Skopje, Skopje, North Macedonia; 15 Faculty of Medicine, University of São Paulo, São Paulo, Brazil; 16 Institute of Inflammation and Ageing, University of Birmingham, B15 2TT Birmingham, UK

**Keywords:** Rehabilitation, Chronic obstructive pulmonary disease

## Abstract

COPD is increasingly common in China but is poorly understood by patients, medications are not used as prescribed and there is no access to recommended non-pharmacological treatment. We explored COPD patients’ and general practitioners’ (GPs) knowledge of COPD, views on its management and the acceptability of a flexible lung health service (LHS) offering health education, exercise, self-management, smoking cessation and mental health support. Using a convergent mixed methods design, data were collected from patients and GPs using focus groups (FGs) in four Chinese cities, questionnaires were also used to collect data from patients. FGs were audio-recorded and transcribed. Quantitative data were analysed descriptively, thematic framework analysis was used for the qualitative data. Two-hundred fifty-one patients completed the questionnaire; 39 patients and 30 GPs participated in ten separate FGs. Three overarching themes were identified: patients’ lack of knowledge/understanding of COPD, current management of COPD not meeting patients’ needs and LHS design, which was well received by patients and GPs. Participants wanted COPD education, TaiChi, psychological support and WeChat for social support. 39% of survey responders did not know what to do when their breathing worsened and 24% did not know how to use their inhalers. 36% of survey respondents requested guided relaxation. Overall, participants did not fully understand the implications of COPD and current treatment was sub-optimal. There was support for developing a culturally appropriate intervention meeting Chinese patients’ needs, health beliefs, and local healthcare delivery. Further research should explore the feasibility of such a service.

## Introduction

As a leading cause of disability and mortality, Chronic Obstructive Pulmonary Disease (COPD) is a significant global public health challenge^1^. In China, where smoking prevalence is high^2^, COPD is the third most common cause of death with a prevalence of 13·7% in those aged 40 years or older^3^. Poor management results in frequent exacerbations and hospitalisation, and therefore high medical costs. In China patients can spend from 33–118% of their average annual income on medical bills, and medical costs to the state attributable to COPD are estimated at ￥195.6 ($30.3) billion, nearly 10% of China’s total health expenditure^4^.

Globally recognised guidelines^5^ recommend tailored treatment for COPD based on symptom profile using a combination of pharmacological and behavioural interventions such as self-management, smoking cessation support or pulmonary rehabilitation (PR)^6^. Despite being shown to be an effective and cost-effective therapeutic strategy^7^, access to PR is problematic due to cost, staff limitations, equipment and widespread lack of GP interest in offering PR, regardless of level of countries’ development^8–11^.

Research indicates that Chinese patients with COPD do not use their medications regularly, have poor understanding of their disease and less than a third participate in regular exercise^3,12–14^. In addition, adherence to PR in trials has been poor^15,16^. Research among healthcare professionals in China shows that prescribing does not always follow international guidelines, knowledge of COPD and PR is poor and PR is not routinely offered^17–20^. However, self-management programmes involving home exercise^21^ and information about COPD and its management in community settings have been shown to be effective in research studies^22^.

PR originated in the West, and is therefore influenced by Western culture. The poor uptake of PR in China may be influenced by local values and beliefs^15,16,23^, which are supported by the inclusion of traditional Chinese medicine (TCM) departments in state hospitals. Therefore, alternative, integrated treatments need to be investigated. A lung health service (LHS) is a more flexible concept, which could be tailored to local need and might include education, exercise, self-management, smoking cessation and psychological support. Involving patients and practitioners in the design of a LHS could help to reduce barriers and improve participation.

Through links with the International Primary Care Respiratory Group, Peking University First Hospital joined with the University of Birmingham (UoB) NIHR Global Health Research Group on Global COPD in Primary Care, “Breathe Well”, to conduct a mixed methods study aiming to explore COPD patients’ and GPs’ knowledge of COPD, their views on its management and the acceptability of a LHS. This was conducted in four primary care community health centres (CHCs) in four different cities in China: Beijing (North), Chengdu (Southwest), Guangzhou (South) and Shenyang (Northeast).

## Methods

This convergent mixed methods study using quantitative (questionnaires) and qualitative focus groups (FGs) methods was conducted from February 2019 to June 2020 with ethical approval from the Ethics Review Board at Peking University First Hospital [No: 2019/52, 13/03/2019] and the Internal Review Ethics Committee at University of Birmingham [IREC2018/1413420, 11/11/2019].

### Participants and recruitment

Patients from urban CHCs in Beijing, Chengdu, Guangzhou and Shenyang, China with a spirometry-diagnosed COPD, and GPs with experience of managing COPD were eligible. We excluded those unable to give informed consent, to speak Mandarin or dialect of, and/or to travel to the FGs. Eligible patients were identified through hospital attendance records and through local GP lists. Researchers then telephoned potential participants to invite them to participate. Procedures for consent are explained below. All participants were free to withdraw at any time, but they were informed that their contributions could not be retrospectively removed from the FG analysis. The electronic data was archived to disc at the end of the study period (25.05.21) and will be destroyed after 5 years along with the paper consent forms.

### Data collection (i): Questionnaires

We assessed patients’ knowledge and understanding of lung disease, identified their current treatment and explored their opinions on a future LHS using a self-constructed questionnaire incorporating validated questionnaires where available to assess outcomes. This included the Lung Information Needs Questionnaire^24^ to describe patients’ knowledge and understanding of their condition, the mMRC Questionnaire^25^ to measure breathlessness and the CAT^26^ to determine patients’ disease-specific health status. After data collection in Beijing was completed, the questionnaire design was reviewed and four additional questions added including the Godin-Shephard Leisure-time Physical Activity questionnaire^27^ to establish current physical exercise status (see Supplementary patient questionnaire). Patient consent was obtained and questionnaires completed when patients were next in attendance at the hospital. Patients participating in the FGs completed the questionnaires prior to the FG to avoid being influenced by other patients. Data were then entered onto REDCap^28,29^.

### Sample size and statistical analysis

No formal sample size calculation was undertaken for this study but a pragmatic approach was taken to collect a representative sample of 100 patients in Beijing and 50 patients in each of the other three cities, around 250 in total. The primary outcome was treatment received by patients, reported as percentages of respondents. The secondary outcomes were patients’ knowledge of the disease and their preference for different treatment options. Descriptive statistics were conducted for all recorded variables using STATA v 15.1^30^. For some questions, where data were missing, pragmatic assumptions were made in order to include the data in Supplementary Table 5.

### Data collection (ii): Focus groups

We conducted two patient and two GP FGs with six to eight participants in Beijing; and one of each in the other three cities. Purposive sampling^31^ was used to ensure that all patient groups included at least one female, and as wide a mix of mMRC dyspnoea scores^25^ as possible. Prior to the COVID-19 pandemic, FGs in the first three cities were conducted face-to-face and written consent obtained on the day of the FG.

In response to COVID-19, FGs in Chengdu were conducted online using the Tencent Conference application, a popular conferencing platform in China, with video functioning. Written consent was obtained in the hospital, at participants’ convenience prior to the day of the FG. Structured topic guides (see Supplementary Topic guides) were used and refined iteratively as themes emerged. All FGs were audio-recorded and transcribed intelligent verbatim. Transcripts were anonymised. During the FG observational notes were taken by the co-facilitator (ZP).

### Qualitative analysis

The transcribed FGs were independently coded by the FG facilitators (HP, ZP and ED) and discussed with the wider research team (RLA and NG). Definitions for each category and subcategory were developed during the process of coding and recorded in a coding diary. Once a coding framework had been agreed it was applied to the remainder of the FGs. The team met to critically reflect on and review interpretations throughout. The data were summarised in Excel using the Framework Method^32^ and themes identified. The analysis reported here focuses on data related to the development of a LHS.

### Interpretation and synthesis of qualitative and quantitative data

The quantitative and qualitative data sets were analysed separately and independently from one another. Once both initial results were available, the lead researcher identified content areas represented in both data sets. The findings were then compared and contrasted in a table to aid understanding of perspectives from patients and GPs.

As findings emerged, the team discussed the extent to which, and in what ways, results from the two types of data converged and related to each other so as to produce a more complete understanding. Thus, both qualitative and quantitative data were used to answer our research question^33,34^.

## Results

### Flow of survey participants

Of 278 eligible COPD patients identified and invited, 23 declined to attend CHCs because of COVID-19 (see Supplementary Fig. 1). Of the 255 patients who provided consent, 4 withdrew because they did not have enough time to complete the questionnaire. In all, 251 patients completed the questionnaires.

### Baseline characteristics

Baseline characteristics are given in Supplementary Table 1. Survey respondents had a mean age of 67.9 (SD 9.1) years, 183 (72.9%) were male, only 64 (25.5%) were current smokers. The majority had mild to moderate COPD with a range of co-morbidities, the top three being hypertension, asthma, and heart disease.

Thirty-nine patients (27 male) and 30 GPs (6 male) participated in ten separate FGs. FG patients had a mean age of 66.3 (SD 7.8) years and 23 patients had a COPD Assessment Test (CAT) score^26^ of 2 to 4, only 10 (25.6%) were current smokers. The majority of the GPs (20, 66.7%) had over 10 years clinical experience.

### Study findings

#### Patients’ knowledge of COPD

Supplementary Table 2 (Questions 1–5) reports patients’ responses regarding knowledge/understanding of COPD. In summary, patients reported not receiving enough advice from their doctors. Over 50% said that there was no advice on how much or what kind of exercise they should do and over 40% no advice on diet, prognosis, breathlessness and management of exacerbations. The main causes of COPD were perceived as smoking, second-hand smoke and air pollution, and the main exacerbation triggers as colds/coughs/flu, air pollution, gases, fumes and dust at work and own or others’ smoking.

#### Current management of lung condition

Supplementary Table 2 (Questions 6–10) reports patients’ report of current management of COPD, their leisure-time exercise (LTE) and affordability of prescribed COPD medication. Patients had been prescribed different treatment options, of which long acting inhalers, oral medication/tablets and short acting bronchodilators were the most common. Up to 39.0% did not know what to do when breathing worsened and 24.3% did not know how to use prescribed inhalers. The majority of the subgroup of patients in Chengdu, Guangzhou and Shenyang were “moderately active” or “active”^27^. Only 42.6% could afford all their treatment.

#### Acceptability of a LHS to patients

Patient views regarding a LHS can be seen in Supplementary Table 3. In summary, the idea of the LHS was well received by patients. The top five preferred components were practicing breathing techniques and advice on: breathlessness, diet, education about lung condition and self-management techniques. Few prioritised stopping smoking, exercise, or psychological counselling. Distance from home was the most important factor influencing their decision to attend a LHS, followed by cost and ease of travel. Over 80% of the subgroup of patients would not pay more than ￥10 ($1.5), equivalent to the cost of five bus rides.

There were 3 main themes (Supplementary Fig. 2) identified from the focus groups:

#### Theme 1: Knowledge/understanding and experience of COPD

Patients were unfamiliar with symptoms of COPD, attributing them, for example, to heart disease and age and not seeking appropriate treatment

*Over ten years ago, I felt tired all the time and my heart beat rapidly so I thought there was something wrong with my heart… I had a coronary angiography but found my heart very good [patient did not take any further action]. [P1, FG3]*

*I had always been in good health so did not pay attention to it [breathlessness]. [P9, FG1]*

Patients lacked understanding about COPD medications. They were worried about dependence and side effects, so did not use them regularly.

*I have an oxygen cylinder at home, but I seldom use it unless I have a fever or catch a cold…If I always passively inhale oxygen, then I might be unable to breathe by myself. That would be terrible. [P7, FG5]*

*I feel better after using my inhalers. But I’m afraid of becoming reliant on them, so stopped them. [P2, FG5]*

Patients reported the negative impact of COPD on sleep, daily activities, memory, ability to work and mood, e.g., depression and short temperedness. They also reported the effect of weather on symptoms. The impact of COPD was worse in young patients and those with severe COPD.

*Sometimes I feel uncomfortable and keep coughing at night, which makes me unable to fall asleep. The next day…I get angry with my family members because of small matters. [P2, FG1]*

*When I go to the south, where the air is moist, my throat becomes better and I rarely cough. [P17, FG2]*

*Every morning I must open the window, or I cannot breathe… I am very afraid when short of breath. [P9, FG5]*

Both GPs and patients reported the burden COPD placed on families.

*He [patient] cannot go out…His wife does everything…All he can do is to ask others [to do things]. So for his partner or caregiver, it may be a huge burden, economic burden. [GP3, FG4]*

*I am interested in photography …but this winter, I was very careful not to catch a cold…my children need to work, my husband is over 70…how could I trouble them? [P7, FG1]*

#### Theme 2: Current management of COPD

Inhalers and lung function tests (LFTs) were rarely available in CHCs, so COPD was not assessed or treated locally. Therefore, GPs received minimal relevant training and lacked confidence in the management of COPD. A local LHS was deemed more effective if run alongside free COPD treatments and tests

*We really hope to have medications recommended by guidelines…we have no medications to prescribe to patients so we do not know their effectiveness, which limits our ability to diagnose and provide treatment. [GP1, FG5]*

*Some doctors do not even know how to use the devices [inhalers] so GPs need education on COPD too. [GP9, FG2]*

*I think they would like to go to big hospitals, but I’m not sure if they would come here. If we can provide free medical tests, such as lung function tests, or pills, gifts, they will be more likely to come. [GP4, FG5]*

Similarly, there were no smoking cessation clinics or cessation drugs in CHCs. While some GPs were happy to recommend specialist cessation advice, others doubted the efficacy of cessation drugs, believing that motivation and peer support were the most effective tools. These latter views were seen in patients too.

*I do not know the effectiveness [of cessation drugs] …What if patients quit smoking but become addicted to smoking cessation drugs? [GP6, FG4]*

*If patients have no motivation to stop smoking, they will not follow advice from their doctors at all. However, if they have the motivation, you [doctors] only need to say a few words and then they quit. [GP2, FG1]*

*I do not want to use cessation drugs because relying on willpower is okay. But most people have not quit because they would change a lot after quitting…they will be fat once quit. (smoker) [P2, FG5]*

Only a few patients visited the TCM department for COPD and held mixed opinions. A couple felt it was useless while another couple found it controlled phlegm well.

*TCM has a drug called “Sanfu patch”* [a kind of patch applied to acupuncture points]*, which is used to treat chronic bronchitis. I used it several times but it did not work. [P3, FG1]*

*I took TCM for nearly half a year and my phlegm stopped. [P14, FG2]*

#### Theme 3: Lung Health Service Design

Subtheme 3.1: COPD education: GPs thought that patients were uninterested in knowing more about COPD, but actually, patients stated that they wanted to learn more

*Our team organised several COPD talks this year but patients were not enthusiastic at all…It was because that they did not think COPD was important. [GP4, FG4]*

*When I visit the outpatient clinic, treatment is a secondary thing. I just want to consult [health information], listen to advice from doctors to improve my knowledge. [P13, FG2]*

GPs were too busy to deliver COPD education, but thought nurses might be able to do so since they had more experience in this area. Both patients and GPs favoured a 1-hour session, once or twice a week.

*Nurses might be more appropriate to give talks since they are more experienced in delivering education than doctors. [GP5, FG1]*

*Twice a week would be appropriate. Sometimes delivering talks on specific topics would be good, and sometimes interactions would be fine. [P7, FG4]*

Some GPs suggested peer education as patients seemed to enjoy meeting with each other and exchanging experiences.

*Some patients I meet would trust their neighbours more [compared with doctors]. When they first attend CHCs, you [GPs] may tell them a lot, but the effectiveness is not as good as what their neighbours said. [GP7, FG2]*

WeChat, an extremely popular social media app in China, has potential for supporting patients.

*During COVID-19 pandemic, it is not feasible to bring patients together so health education is mostly done online…Many people messaged me to say that this form is quite good. [GP4, FG3]*

*Patients with COPD could be in the same [WeChat] group to share our medications and experience in treating COPD. This would be great. [P2, FG5]*

*Subtheme 3.2: Exercise training:* GPs were concerned about the risk of injuries from exercise training, and subsequent litigation so wanted written consent beforehand.

*If we want to get PR to work, patients need to do intensive exercise, then there will be risks…patients’ written consent is insufficient, their children need to consent, too. [GP1, FG1]*

Some GPs felt that they had insufficient time or knowledge to deliver exercise training so suggested specialists as an alternative. However, those with experience in teaching TaiChi were keen to receive more training so they could deliver this themselves.

*If delivering PR in CHCs, the first problem would be personnel. We could not ask clinicians to do this because they are unable to do it well. I think we could recruit some rehabilitation doctors and physiotherapists. [GP5, FG3]*

*I advise patients to do TaiChi… A few days ago, we worked with the TCM Department to bring together a dozen COPD patients to do Liuzijue [a moderate-low intensity form of traditional Chinese exercise]…patients are happy to learn it and our medical staff need to do more [to be able to help them]. [GP1, FG4]*

While patients with mild COPD were agreeable to the idea of strenuous exercise, others were not.

*I think that I would become more breathless if taking exercise…Anyway, I cannot take strenuous exercise but mild exercise might be acceptable. [P3, FG5]*

*Chinese people are not as strong as foreigners and many elderly people have co-morbidities. [GP3, FG1]*

*Subtheme 3.3: Psychological support:* There were mixed views about the management of psychological wellbeing. Some GPs and patients felt that better education about COPD would alleviate patient anxiety, while more formalised psychological support would need specialists.

*I would not feel anxious if I understand the disease. [P9, FG5]*

*I think the best way is to let them understand that COPD can be prevented and controlled. Then they will not be worried anymore. [GP4, FG3]*

*I provided psychological counselling before, but it was not useful. I think it is better to provide psychological support from specialists. After all, I’m not good at that. [GP1, FG4]*

Integration of the survey and FG findings can be seen in Supplementary Table 4.

## Discussion

We found that participants did not fully understand the implications of COPD and how best to manage it. Current health service availability did not meet patients’ needs and for some was too costly. TCM was not as important to patients as expected. There was support for a LHS among both COPD patients and GPs, one tailored to meet Chinese patients’ needs, health beliefs and local healthcare delivery, e.g., type of exercise, patient led topics, user-friendly appointments and use of WeChat. Patients wanted a flexible service, GPs suggested that nurses and physiotherapists would be best placed to provide this. There were concerns about safe and acceptable levels of exercise; TaiChi and walking might be fitting alternatives to other forms of exercise. Psychological support was not considered necessary by patients or GPs.

This study used questionnaires and FGs to gather data from both patient and GP perspectives to inform the design of a flexible and culturally appropriate intervention. The triangulation of data from different methods enabled the team to access the perspectives of different groups, for instance, those with severe COPD were not able to participate in FGs, but were able to engage with the survey. The survey also offered anonymity for the research participants enabling the team to engage with challenging topics such as finance, whereas those in FGs denied any concerns about being able to afford their healthcare. Either this was a skewed sample or participants did not feel comfortable sharing financial difficulties. Recruitment sites represented different geographic areas of China, where exposure, lifestyle and the prevalence of COPD differed, enriching the data’s diversity.

There were some limitations to the study. In some cases, the research team felt that qualitative responses lacked depth or explanation, such as in the online FGs where patient participation was much lower than face-to-face, potentially due to unstable network connections and the mode of communication, or where facilitators were trained but not experienced in FG facilitation, so did not probe further. Study participants were limited to provincial capital cities, which may affect the external validity of the study. In addition, variable recall may have affected participants’ responses. In order to maximise diversity within the qualitative data we purposively sampled different kinds of people and analysed the data alongside collection to ensure data saturation, that is, the point at which no new themes were emerging^31^. A larger survey sample could potentially have provided the opportunity to examine whether the results differed by population subgroups.

In line with previous research^3,35–37^, we found that COPD was poorly understood by patients and that current management was sub-optimal with GPs having limited clinical skills in this area^38^. A potential solution suggested in studies from Europe, which is also emphasised in China’s recent government policies, is to develop postgraduate training programmes, which address the value of PR in supporting the management of COPD in primary care^36,39,40^.

A systematic review comparing PR programmes globally revealed that the current availability of PR services among individuals with COPD was less than 1.2%^41^. The importance of offering a flexible tailored service was highlighted, failure to do so may explain why some services experience low uptake^13^. In addition to providing user-friendly appointments, the use of novel modes of delivery and culturally acceptable elements are important factors - in 2019 1.15 billion users a month logged onto WeChat, a social media app popular in China^42^. This app is known to be effective in delivering health education, remote intervention and follow-up^43–48^. Favourable comments about WeChat’s potential for COPD education were made by both patients and GPs in our study. The poor inhaler technique identified in this study is a global issue with little improvement over the past 40 years^49^. Video applications used to monitor patients’ inhaler techniques at home have found that errors in technique start appearing as early as the first day after training^50^. WeChat might be a useful tool for improving inhaler technique.

In keeping with other studies in China and also America^51,52^, patients in our study were reluctant to take strenuous exercise due to shortness of breath, gentle exercise like TaiChi, QiGong and walking were considered more appropriate^51^. However, it should be recognised that they may not be as effective in building endurance and strength as other forms of exercise usually used in PR^53–58^. A new finding was GPs’ concern about litigation. These concerns may be explained by recent research in China reporting increasing litigation in medicine, some of which have resulted in, verbal or physical abuse towards doctors^59,60^.

These findings can be used to design a feasibility study testing a culturally appropriate LHS, including education on COPD, exercise, diet, oxygen therapy and medication use, which contrary to GP beliefs, patients are likely to be receptive to. The lack of lung function testing and COPD drugs in community health centres limits GPs’ ability to identify and therefore treat COPD locally, negatively impacting on their expertise in this area. Preliminary evidence demonstrates the ease with which this could be achieved within community settings in China^61^. If government policy regarding COPD is to be more effectively implemented these limitations need addressing. The burden imposed upon family members by patients’ exacerbations and hospitalisations also needs to be considered and addressed, similarly a review of healthcare costs could further improve COPD patients’ health. Further work could explore acceptability in rural locations, which was not directly addressed in this study. In addition to the issues regarding provision of PR services, not just in China, but globally^41^, uptake is also a factor. The suggestion in our study that this may be due to design issues, which do not meet local needs emphasises the benefits of studies like ours being conducted in other countries, as locally tailored, flexible LHS’s might be more culturally acceptable.

Overall, this study showed that participants did not fully understand the implications of COPD and current treatment was sub-optimal. There was support for developing a culturally appropriate intervention among COPD patients, one tailored to meet Chinese patients’ needs, health beliefs, and local healthcare delivery. TaiChi was suggested as an acceptable intervention with WeChat for social support. Further research is needed to explore the feasibility of such a service.

### Reporting summary

Further information on research design is available in the Nature Research Reporting Summary linked to this article.

## Supplementary information

Supplementary information file

REPORTING SUMMARY

## Data Availability

The data supporting the findings of this study are available from the corresponding author upon written application and completion of appropriate data sharing agreements.
