# Supplementary material for: Community lung health service design for COPD patients in China by the Breathe Well group
Source: NPJ Prim Care Respir Med. 2022 Aug 19;32:27. doi: 10.1038/s41533-022-00286-8 (PMC9388970; doi:10.1038/s41533-022-00286-8)
Supplement: Supplementary file 1 — Supplementary information file [file 41533_2022_286_MOESM1_ESM.docx]

Supplementary Figure 1 Flow of survey participants

Supplementary Table 1 Participant characteristics

Supplementary Table 2 Patients’ knowledge & understanding of COPD and current management

Supplementary Table 3 Design of Lung Health Service

Supplementary Figure 2 Summary of themes discussed by participants

Supplementary Table 4 Integration of survey and qualitative results

Supplementary Patient questionnaire

Supplementary Table 5 Missing data

Supplementary Topic guide-Patients

Supplementary Topic guide-GPs

Eligible patients and those identified from GPs’ lists (N=278) Locations: Beijing (n=98), Chengdu (n=65), Guangzhou (n=57), Shenyang (n=58)

Consented (n= 255)

Declined: n=23 (declined to attend CHCs)

Withdrawals: n=4 (no time to complete questionnaire)

Completed and final analysis (n=251)

Supplementary Figure 1 Flow of survey participants

| **Supplementary Table 1 Participant characteristics** | | | |
| --- | --- | --- | --- |
|  | **Questionnaire**  **n (%)** | **Patient FGs**  **n (%)** | **GP FGs**  **n (%)** |
| **Total participants** | **N=251** | **N=39** | **N=30** |
| Male | 183 (72.9) | 27 (69.2) | 6 (20.0) |
| **Age in yrs, mean (SD)** | 67.9 (9.1) | 66.3 (7.8) | 39.5 (8.0) |
| **Education** | |  |  |
| No formal/less than high school | 124 (49.4) |  |  |
| High school | 84 (33.5) |  |  |
| Bachelor/Master | 43 (17.1) |  |  |
| **Live alone** | 33 (13.2) |  |  |
| **Live in urban area** | 227 (90.4) |  |  |
| **Employment status** | |  |  |
| Employed | 24 (9.6) | 3 (7.7) | 30 (100.0) |
| Unemployed | 19 (7.6) | 1 (2.6) | 0 (0.0) |
| Retired | 208 (82.9) | 35 (89.7) | 0 (0.0) |
| **Current smoking status** | |  |  |
| Current smoker | 64 (25.5) | 10 (25.6) |  |
| Ex-smoker | 103 (41.0) | 13 (33.3) |  |
| Never smoked regularly | 84 (33.5) | 16 (41.0) |  |
| **Pack years, mean (SD)** | 35.3 (32.6) |  |  |
| **Electronic cigarettes use (current/ex/never)** | 0 |  |  |
| **Health in general** | |  |  |
| Very good/good | 67 (26.7) |  |  |
| Fair | 122 (48.6) |  |  |
| Bad/very bad | 61 (24.3) |  |  |
| Missing | 1 (0.4) |  |  |
| **Co-morbidities^a^** | |  |  |
| Hypertension | 103 (41.0) |  |  |
| Asthma | 78 (31.1) |  |  |
| Heart disease | 66 (26.3) |  |  |
| Other^b^ | 75 (29.9) |  |  |
| None of above | 3 (1.2) |  |  |
| **Modified British Medical Research Council (mMRC)**^24^ | |  |  |
| Grade 0-1 | 157 (62.5) | 16 (41.0) |  |
| Grade 2-4 | 94 (37.5) | 23 (59.0) |  |
| **CAT, mean (SD)** | 13.3 (8.5) |  |  |
| **Years of experience** |  |  |  |
| 0-9 |  |  | 10 (33.3) |
| ≥10 |  |  | 20 (66.7) |
| **Position** |  |  |  |
| Physician or below |  |  | 19 (63.3) |
| Deputy chief physician and above |  |  | 11 (36.7) |
| **FG** Focus group  **COPD** Chronic Obstructive Pulmonary Disease  **^a^** Participants could select more than one response  ^b^ Diabetes Mellitus (39, 15.5%), GERD or Reflux (25, 10.0%), Anxiety (13, 5.18%), Tuberculosis (11, 4.4%), Cancer (8, 3.2%), Depression (6, 2.4%) | | | |

| **Supplementary Table 2 Patients’ knowledge/understanding of COPD and current management** | | |
| --- | --- | --- |
| Question area | **Response Options** | **n/N (%)** |
| 1 Knowledge of lung condition’s name | Yes | 207/251 (82.5) |
|  | No | 40/251 (15.9) |
|  | Missing | 4/251 (1.6) |
| 2 Expectation of condition’s progression over next few years | Get better | 43/251 (17.1) |
|  | Stay the same | 106/251 (42.2) |
|  | Get worse | 55/251 (21.9) |
|  | I don't know | 45/251 (17.9) |
|  | Missing | 2/251 (0.8) |
| 3 Understanding of lung condition & advice received from doctor |  | **Yes** |
|  | I understand lung condition | 182/251 (72.5) |
|  | My doctor has told me what is likely to happen in the future | 127/251 (50.6) |
|  | My doctor has told me what to do if I become breathless | 133/251 (53.0) |
|  | My doctor has told me what to do if I have an exacerbation | 131/251 (52.2) |
|  | My doctor has shown me how to take my medication | 186/251 (74.1) |
|  | My doctor has advised me (current/ex smoker) to stop smoking | 123/167 (73.7) |
|  | My doctor has advised me (current/ex smoker) on how to stop smoking | 76/167 (45.5) |
|  | My doctor has advised me on what I should eat | 12/251 (48.6) |
|  | My doctor has advised me on how much exercise I should do | 108/251 (43.0) |
|  | My doctor has advised me on what kind of exercise I should do | 109/251 (43.4) |
| 4 Perceived importance of factors in causing lung condition |  | **Important/ very important** |
|  | Colds/Coughs/Flu | 129/251 (51.4) |
|  | My smoking | 159/251 (63.4) |
|  | Second hand Smoke | 153/251 (61.0) |
|  | Gases, fumes or dust at work | 139/251 (55.4) |
|  | Cooking Fumes | 117/251 (46.6) |
|  | Biofuels | 121/251 (48.2) |
|  | Air pollution | 153/251 (61.0) |
|  | Other^c^ | 18/251 (7.2) |
| 5 Perceived importance of causal factors for exacerbations |  | **Important/ very important** |
|  | Cold/Coughs/Flu | 179/251 (71.3) |
|  | My smoking | 158/251 (63.0) |
|  | Second hand Smoke | 182/251 (64.5) |
|  | Gases, fumes or dust at work | 163/251 (64.9) |
|  | Cooking Fumes | 127/251 (50.6) |
|  | Air pollution | 164/251 (65.3) |
|  | Other^d^ | 4/251 (1.6) |
| 6 When COPD was diagnosed (n=155)^e^ | In the last year | 68/155 (43.9) |
|  | In the last 5 years | 36/155 (23.2) |
|  | In the last 10 years | 15/155 (9.7) |
|  | More than 10 years ago | 35/155 (22.6) |
|  | Missing | 1/155 (0.6) |
| 7 Current management of lung condition |  | **Yes** |
|  | I know where to go to get treatment for my lung condition | 196/251 (78.1) |
|  | I can easily travel to the hospital^f^ | 221/251 (88.1) |
|  | I know how to use my inhalers | 163/251 (64.9) |
|  | I know how to take my medication correctly | 175/251 (69.7) |
|  | I know what to do when my breathing gets worse | 142/251 (56.6) |
| 8 Prescribed treatment^a^ | Long acting inhalers | 144/251 (57.4) |
|  | Short acting bronchodilators | 73/251 (29.1) |
|  | Oral medication / tablets | 115/251 (45.8) |
|  | Antibiotics | 49/251 (19.5) |
|  | Traditional Chinese medicines | 47/251 (18.7) |
|  | Supervised exercise programme | 15/251 (6.0) |
|  | Breathing exercises | 63/251 (25.1) |
|  | Other^g^ | 3/251 (1.2) |
| 9 Reported level of physical activity (based on responses to Godin-Shephard Leisure-Time Physical Activity Questionnaire (n=155)^e^ | Insufficiently active/sedentary | 23/155 (14.8) |
|  | Moderately active | 92/155 (59.4) |
|  | Active^h^ | 38/155 (24.5) |
|  | Missing | 2/155 (1.3) |
| 10 Affordability of prescribed treatment for lung condition | No, I cannot afford it | 30/251 (12.0) |
|  | I can afford some of it | 112/251 (44.6) |
|  | Yes, I can afford all of it | 107/251 (42.6) |
|  | Missing | 2/251 (0.8) |
| **^a^** Participants could select more than one response.  **^c^** Gene, heart disease, rhinitis, etc.  **^d^** Chemical fume, activity, exercise & benzene-containing compound.  **^e^** New question added for Chengdu, Guangzhou & Shenyang.  ^f^ CHC v tertiary hospital^^[[1]](#footnote-1)^^ not specified in questionnaire.  **^g^** Walking & swimming. | | |

| **Supplementary Table 3 Design of Lung Health Service** | | |
| --- | --- | --- |
| **Question area** | **Response options** | **n/N (%)** |
| 11 Interest in taking part in a LHS | Yes | 131/251 (52.2) |
|  | Possibly | 69 /251 (27.5) |
|  | No | 50/251 (20.0) |
|  | Missing | 1/251 (0.4) |
| 12 Preferred time for LHS^a^ | Morning (Mon-Fri) | 116/251 (46.2) |
|  | Afternoon (Mon-Fri) | 87/251 (34.7) |
|  | Evening (Mon-Fri) | 47/251 (18.7) |
|  | Weekends (Beijing only) (n=96) | 40/96 (41.7) |
|  | Weekends morning (n=155)^e^ | 27/155 (17.4) |
|  | Weekends afternoon (n=155)^e^ | 23/155 (14.8) |
|  | Weekends evening (n=155)^e^ | 16/155 (10.3) |
| 13 Preferred number of sessions^a^ | 1 session a week for 8 to 12 weeks | 74/251 (29.5) |
|  | 2 sessions a week for 6 to 8 weeks | 41/251 (16.3) |
|  | 1 session a week for 6 to 8 weeks | 90/251 (35.9) |
|  | 2 sessions a week for less than 6 weeks | 29/251 (11.6) |
| 14 Preferred form of LHS (1:1 or group) | I start the programme in a group and we attend the same sessions each week | 53/251 (21.1) |
|  | I start the programme when it is convenient for me but attend the same session each week | 67/251 (26.7) |
|  | I attend the most convenient session each week | 79/251 (31.5) |
|  | I complete the programme by myself | 29/251 (11.6) |
|  | Missing | 23/251 (9.2) |
| 15 Preferred location for LHS | Main Hospital | 18/251 (7.2) |
|  | Community health centres | 163/251 (64.9) |
|  | My Home | 42/251 (16.7) |
|  | Other^i^ | 5/251 (2.0) |
|  | Missing | 23/251 (9.2) |
| 16 Top five preferences for content of LHS | Breathing techniques | 170/251 (67.7) |
|  | Advice about what to do if I become suddenly breathless | 139/251 (55.4) |
|  | Advice about what I should eat | 116/251 (46.2) |
|  | Education about my lung condition | 115/251 (45.8) |
|  | Self-Management Techniques | 107/251 (42.6) |
|  | Guided relaxation exercise (TaiChi, Qi Gong) | 90/251 (35.9) |
|  | Inhaler technique advice | 78/251 (31.1) |
|  | Support and advice for my family and carers | 74/251 (29.5) |
|  | General advice about what exercise I should do | 69/251 (27.5) |
|  | Supervised aerobic exercise | 48/251 (19.1) |
|  | Advice about stopping smoking | 32/251 (12.8) |
|  | Supervised exercises with weights | 29/251 (11.6) |
|  | Advice about coping with work and my lung condition | 23/251 (9.2) |
|  | Counselling for anxiety/depression | 12/251 (4.8) |
| 17 Preference regarding exercise supervision (n=155)^e^ | With supervision | 92/155 (59.4) |
|  | Without supervision | 17/155 (11.0) |
|  | Both is okay | 40/155 (25.8) |
|  | Missing | 6/155 (3.9) |
| 18 Top three most important factors influencing decision to attend LHS | How far away I live | 147/251 (58.6) |
|  | If I had to pay for the sessions | 110/251 (43.8) |
|  | How easy it is to travel to the sessions | 98/251 (39.0) |
|  | If the sessions meant I missed work or other important activities | 89/251 (35.5) |
|  | How many weeks the programme lasts | 57/251 (22.7) |
|  | If my doctor recommended the programme | 53/251 (21.1) |
|  | The type of exercise or activity involved | 50/251 (19.9) |
|  | How many sessions per week | 37/251 (14.7) |
|  | If someone I knew was also participating | 4/251 (1.6) |
| 19 Appropriate costs for LHS (n=155)^e^ | <10 RMB (<$1.5) | 104/155 (67.1) |
|  | 10-50 RMB ($1.5-7.5) | 15/155 (14.3) |
|  | 50-100 RMB ($7.5-15) | 3/155 (2.0) |
|  | 100-200 RMB ($15-30) | 1/155 (0.7) |
|  | Others (Free of charge) | 27/155 (17.4) |
|  | Missing | 5/155 (3.2) |
| **^a^** Patients could select more than one response.  **^e^** New question added for Chengdu, Guangzhou & Shenyang.  **^i^** Finish it by myself, in the park or nursing home.  **CHC** community healthcare centre. | | |

**Exercise training**

Litigation concerns

**COPD education**

**Psychological support**

Patients unfamiliar with symptoms of COPD

Poor patient understanding of medications

Negative impact of COPD on patients

Emotional and economic burden for families

**Knowledge and experience**

**Lung Health Service**

CHCs need inhalers to treat COPD locally and maintain skills

Benefits of cessation drugs and behavioural support poorly understood

TCM not widely used

Patients want more information

Nurses to deliver 1-hour session, once/twice a week

Consider peer education

WeChat

COPD education may improve psychological well being

Use specialists

Mild exercise favoured, e.g. TaiChi

**Current management**

GPs lack confidence managing COPD, want more training

Supplementary Figure 2 Summary of themes discussed by participants.

| **Supplementary Table 4 Integration of survey and qualitative results** | | | |
| --- | --- | --- | --- |
| **DATA** |  |  | **INTEGRATION** |
| **Summary from Survey** | **Summary from Patient FGs** | **Summary from GP FGs** | **LHS Implications** |
| **Knowledge/understanding and experience of COPD** |  |  |  |
| *1 Knowledge of the label “COPD”* |  |  |  |
| Nearly a fifth did not know they had COPD | Patients unable to remember information and wanted more. | Patients cannot remember their diagnosis. | **Patient education to explore what patients want at diagnosis and in what format (written material atypical in China and average reading age in this population extremely low), consider including family without overburdening them.** |
| *2 Perceived prognosis* |  |  |  |
| Only a fifth thought it would get worse | Patients did not know their prognosis. | Patients’ knowledge affects adherence and confidence to manage COPD. | **Patient education to explain likely future prognosis.** |
| *3 Understanding of COPD & advice from doctor* |  |  |  |
| Three quarters said they understood their disease, but around half reported receiving no advice on exercise, diet, prognosis, breathlessness or exacerbations | Exercise advice ignored because of breathlessness. Advised to quit smoking, believe willpower is best. | GPs wanted more training to deliver COPD information. Queried usefulness of smoking cessation drugs, saw motivation and peer support as more effective. Some recognised role of specialist cessation support. | **Patient education on living with COPD. GP training on providing advice.** |
| *4* *Importance of factors causing COPD* |  |  |  |
| Most selected cigarette smoke and air pollutants as important | Smoking was key, also noted: chemical agents and genetics. |  | **Patient education about COPD risk factors.** |
| *5 Importance of factors causing exacerbations* |  |  |  |
| Most people considered colds/coughs/flu and air pollutants important. | Weather, tiredness, colds, cooking fumes, cigarette smoke and dust. | Weather - some patients go south in winter. | **Patient education about triggers for exacerbations. Weather may affect LHS attendance.** |
| **Current management of COPD** |  |  |  |
| *6 Time of diagnosis^e^* |  |  |  |
| Over two fifths of subgroup diagnosed in last year | Patients unfamiliar with factors indicative of COPD. | Patients lacked awareness of COPD and screening. Some were initially misdiagnosed in CHCs. | **Public awareness needs increasing. Screening strategies need review.** |
| *7 Current management of COPD* |  |  |  |
| Many did not understand their medication or what to do when breathless | Patients unaware inhaler techniques poor. CHCs preferred for COPD management as closer, cheaper and give better explanations. | Poor patient inhaler techniques and incorrect use of O_2_^k^ at home. Patients preferred tertiary hospitals for COPD management as “better doctors”. | **Patient education on inhaler technique and home O_2_. CHCs preferred for COPD treatment.** |
| *8 Treatment options* |  |  |  |
| Over half used long-acting inhalers, only a few used TCM or supervised exercise | Some saw inhalers as ineffective, tablets trusted more. | Patients trust tablets more as more perceptible. | **Patient education about the purpose of medications** |
| *9 Leisure-time exercise score^e^* |  |  |  |
| Most of the subgroup were moderately active | Every-day walking and daily activities considered enough. | For safety, only mild exercise recommended. | **There may be resistance to increased exercise.** |
| *10 Cost of the treatment* |  |  |  |
| Less than half could afford all their healthcare costs | Costs not a problem as covered by health insurance. | Economic burden of COPD to patients because of hospitalisation. | **LHS could reduce economic burden of exacerbation/ hospitalisation. Offering free COPD treatments and tests as well would increase uptake.** |
| **Lung Health Service Design** |  |  |  |
| *11 Interest in the program* |  |  |  |
| Four fifths were interested in a LHS | Patients interested in talks on COPD and PR. | Patients’ understanding of COPD, education level, efficacy of LHS’s and trust in their doctors would affect uptake. Offering a free COPD service would be an incentive. Numbers of COPD patients registered at CHCs currently low. | **Include these factors in LHS design, consider patient incentives.** |
| *12 Time of programme* |  |  |  |
| Most wanted a weekday | Times need to fit childcare and patients’ exercise routines. | Options need to accommodate patients’ exercise routines, impact of symptoms and weather. | **Provide choice of slots.** |
| *13 Preferred frequency* |  |  |  |
| Most preferred 1 session a week | Once a week or once a fortnight due to travel time to CHCs, childcare and housework. | Once or twice a week. | **Weekly sessions.** |
| *14 Appointment slots* |  |  |  |
| Nearly half wanted to choose a fixed slot that was convenient for them | Some may be unable to attend a fixed slot due to housework and childcare. Some preferred learning via WeChat. | Supported group work but numbers may be a barrier. | **Group education suggested, choice of slots and sharing materials via WeChat.** |
| *15 Location* |  |  |  |
| Most preferred CHCs | CHCs closer but tertiary hospitals had “better doctors”. | CHCs closer but challenges include personnel, funding, space, equipment. | **CHCs preferred, but support needed.** |
| *16 Top 5 preferred components* |  |  |  |
| Advice on breathing techniques, breathlessness, diet, COPD information and self-management. Support with anxiety and depression ranked last | Wanted information on prognosis, treatment, medications, exercise, diet, prevention and home O_2_^l^. Felt that being better informed would alleviate emotional problems. | Diet important. Emotional support and COPD information might alleviate patients’ anxiety. Suggested nurses to deliver education. | **Patient education on living with COPD, this may also improve emotional wellbeing. Involve nurses.** |
| *17 Exercise supervision^e^* |  |  |  |
| Over half the subgroup wanted supervision | Supervision wanted for safety. Walking and TaiChi favoured. | Supervision and written consent needed to reduce litigation, specialists could deliver. Support for TaiChi. | **Supervision and consent important. Involve specialists. TaiChi and walking suggested, but exercise needs to be of sufficient rigour.** |
| *18 Top 3 factors influencing decision to attend* |  |  |  |
| Distance most important, then cost and ease of travel | Distance, cost and effectiveness important. | Distance and costs to patient important. | **Consider distance, travel costs and routes for LHS.** |
| *19 Appropriate costs for a LHS^e^* |  |  |  |
| Max for most of the subgroup was ￥10 ($1.5) per session | Cheaper was better. | Should be free. | **Keep LHS costs low.** |
| **^e^** New question added for Chengdu, Guangzhou & Shenyang.  ^k^ This refers to privately purchased O_2_, O_2_ is not available through health insurance in China so any patient can buy O_2_ for personal use without prescription or health & safety advice.  ^l^ Patients have to travel to tertiary hospitals to renew their inhalers. | | | |

**Supplementary Patient questionnaire**

**探索中国慢阻肺患者对“肺健康”服务项目的需求与方案设计：混合方法研究
Exploring the need for, and design of, a community “lung health” service for COPD patients in China: A Mixed Methods Study**

您的回答和意见对我们很有价值。请您在翻页之前阅读以下内容，非常感谢您的合作！

Your answers and opinions are valuable to us. We would be very grateful if you could read the below before turning the page:

如有可能，请您自行填写这份问卷。

Please complete this questionnaire yourself if at all possible

请尽可能回答所有问题

Please answer all questions as well as you can

请不要花太多时间思考您的回答

Do not spend too long thinking about your answers

如果有人替您回答了这份问卷，他们需要记录下您的答案

If someone is completing this on your behalf, they should record your answers

| Patient ID 病人身份ID |  |
| --- | --- |
| Date 日期 |  |


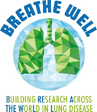


**Part 1**

**1. 性别 - Sex**

| 男 - Male |  |
| --- | --- |
| 女 - Female |  |

**2. 年龄 - What is your age?**

__________ 岁 - years old

**3. 您获得的最高学历是什么？- What is the highest level of qualification that you have?**

| 没有正式的学历 No formal qualification |  |  | 本科 Bachelor |  |
| --- | --- | --- | --- | --- |
| 低于高中水平 Less than High school |  |  | 硕士Master |  |
| 高中水平 High school |  |  | 博士 Doctor |  |

**4. 您是否独居？ Do you live alone?**

| **是** Yes | **否** No |
| --- | --- |

**5. 您绝大部分时间生活在哪里？- Where have you spent most of your life?**

| \| 城市 Urban areas \|  \| \| --- \| --- \| \| 农村 Rural areas \|  \| |
| --- | --- | --- | --- | --- |

**6. 您的工作状态如何？- What is your employment status?**

| 个体 Self-employed |  |
| --- | --- |
| 受雇于单位 Employed |  |
| 无工作 Unemployed |  |
| 退休 Retired |  |

**7. 您目前的吸烟状态是？- What is your current smoking status?**

| 当前吸烟者（每天至少吸1支，至少吸了6个月）  Current smoker (smoke at least 1 cigarette per day for at least the last 6 months) |  |
| --- | --- |
| 既往吸烟者（既往每天至少吸1支，至少吸了6个月，但是现在不吸了）  Ex-smoker (previously smoked at least 1 cigarette per day for at least 6 months, but not now) |  |
| 我从不经常性地吸烟 （如果您选择了这个选项，请跳至第10题）  I have never smoked regularly （*please go to question 10*） |  |

**8. 如果您曾经吸过烟，那么您是几岁开始经常性地吸烟？（“经常性地吸烟”指的是，至少1支/每天或者7支/每周，至少6个月）If you have ever smoked, at about what age did you start to smoke regularly? (by regularly we mean at least 1 cigarette/day or 7 cigarettes/week for at least 6 months)**

_______ 岁

**如果您曾经吸过烟，您是从什么时候停止经常性地吸烟的？If you are an ex-smoker, at what age did you stop smoking regularly?**

_______ 岁

**9．目前您每天常常吸多少支烟？或者，当您是烟民的时候，您是否经常性地吸烟？How much do you usually smoke each day now, or did you usually smoke when you were a smoker?**

| 电子烟 - Electronic cigarettes (or e-cigarettes) |  | 支/天time/day |
| --- | --- | --- |
| 过滤嘴型香烟 - Filter cigarettes |  | 支/天number/day |
| 无过滤嘴/手卷烟 - Non-filter/hand rolled cigarettes |  | 支/天number/day |
| 雪茄 - Cigars |  | 支/天 time/day |
| 烟斗 - Pipe tobacco |  | 支/天 number/day |

**10．您的整体健康状况如何? How is your health in general?**

| 非常好 Very Good |  |
| --- | --- |
| 好 Good |  |
| 一 般 Fair |  |
| 差 Bad |  |
| 非常差 Very Bad |  |

**11．您患有以下疾病吗？请选择 - Has a doctor EVER told you that you had any of the following conditions? Please tick all that apply**

| 疾病 Conditions | Yes |  | Yes |
| --- | --- | --- | --- |
| 慢性阻塞性肺疾病  Chronic Obstructive Pulmonary Disease |  | 癌症  Cancer |  |
| 慢性支气管炎/肺气肿  Chronic bronchitis/emphysema |  | 胃食管返流  GERD or Reflux |  |
| 哮喘  Asthma |  | 焦虑  Anxiety |  |
| 结核  Tuberculosis |  | 抑郁  Depression |  |
| 高血压  Hypertension |  | 心脏病  Heart disease |  |
| 糖尿病  Diabetes Mellitus |  |  |  |

**12. Please circle the best response to describe your shortness of breath:
请圈出描述您呼吸困难程度最合适的等级：**

| **Grade**  **评价等级** | **Description of breathlessness**  **严重程度** |
| --- | --- |
| **0** | 只在剧烈运动时感到呼吸困难  I only become breathless with strenuous exercise |
| **1** | 在快走或上缓坡时感到呼吸困难  I get short of breath when hurrying on the level or walking up a slight hill |
| **2** | 由于呼吸困难，比同龄走得慢，或者以自己的速度在平地上行走时需要停下来呼吸  I walk slower than most people the same age on the level because of breathlessness, or I have to stop for breath when walking at my own pace on the level |
| **3** | 在平地上步行100米或数分钟需要停下来呼吸  I stop for breath after walking about 100 metres or after a few minutes on the level |
| **4** | 因为明显呼吸困难而不能离开房屋或者换衣服时也感到气短  I am too breathless to leave the house or I am breathless when dressing or undressing |

**慢阻肺评估 (CAT)**

**13. 您肺部的问题怎么样？对于下面的每个项目，请在0-5中标记出您最符合的分数。How are your lung problems? For each item below place a mark in the box that best describes your experience on a scale of 0-5**

| 我从不咳嗽  I never cough | 0 1 2 3 4 5 | 我总是咳嗽  I cough all the time |
| --- | --- | --- |
| 我肺里一点痰也没有  I have no phlegm (mucus) in my chest at all | 0 1 2 3 4 5 | 我肺里有很多很多痰  My chest is completely full of phlegm (mucus) |
| 我一点也没有胸闷的感觉  My chest does not feel tight at all | 0 1 2 3 4 5 | 我有很重的胸闷的感觉  My chest feels very tight |
| 当我在爬坡或爬一层楼梯时,  我并不感觉喘不过气来  When I walk up a hill or one flight of stairs I am not breathless | 0 1 2 3 4 5 | 当我在爬坡或爬一层楼梯时,  我感觉非常喘不过气来  When I walk up a hill or one flight of stairs I am very breathless |
| 我在家里的任何活动都不受  慢阻肺的影响  I am not limited doing any activities at home | 0 1 2 3 4 5 | 我在家里的任何活动都很受 , 慢阻肺的影响  I am very limited doing activities at home |
| 尽管我有肺病, 我还是有信心外出  I am confident leaving my home despite my lung condition | 0 1 2 3 4 5 | 因为我有肺病 , 对于外出我完全没有信心  I am not at all confident leaving my home because of my lung condition |
| 我睡得好  I sleep soundly | 0 1 2 3 4 5 | 因为我有肺病，我睡得不好  I don’t sleep soundly because of my lung condition |
| 我精力旺盛  I have lots of energy | 0 1 2 3 4 5 | 我一点精力都没有  I have no energy at all |

COPD评估测试和CAT标识为葛兰素史克集团公司拥有之商标。葛兰素史克集团公司版权所有。保留所有权利

**Part 2**

**1. 您知道您得的是什么肺病吗？ - I know the name of my lung disease**

| **是**  Yes (请在右侧的方框中写出您的肺病的名称) |  |
| --- | --- |
| **否**  No |  |

**2. 您什么时候诊断的慢阻肺?(请选择一项) When were you diagnosed with COPD? (Please select 1).**

| 1年前 In the last year |  |
| --- | --- |
| 5年前 In the last 5 years |  |
| 10年前 In the last 10 years |  |
| 超过10年前 More than 10 years ago |  |

**3. 下面哪一句话能最准确得描述在未来几年里您的身体状况？ 请只选一项Which of the following statements best describes what will happen to you over the next few years? (Tick only one)**

| 现在我正在治疗，所以我的身体状况可能会变好Now that my disease is being treated, I will probably get better |  |
| --- | --- |
| 现在我正在治疗，所以我的身体状况可能会保持稳定  Now that my disease is being treated, I will probably stay the same |  |
| 我的病会加重  I will get worse |  |
| 我不知道  I have no idea |  |

**4.该问题主要关于您对肺部状况的了解以及您从医生处得到的建议。This question is about your understanding of your lung condition and the advice you have received from your doctor.**

|  | 是Yes | 否No | 不适用N/A |
| --- | --- | --- | --- |
| 我了解我慢阻肺的情况 I understand my lung condition |  |  |  |
| 我的主治医生已经告诉了我未来可能会发生的事情  My doctor has told me what is likely to happen in the future |  |  |  |
| 我的主治医生已经告诉我当我感到呼吸困难的时候应该如何做  My doctor has told me what to do if I become breathless |  |  |  |
| 我的主治医生已经告诉我当我病情加重时应该如何做  My doctor has told me what to do if I have an exacerbation |  |  |  |
| 我的主治医生已经向我说明了应该如何服药  My doctor has shown me how to take my medication |  |  |  |
| 我的主治医生已经建议我戒烟  My doctor has advised me to stop smoking |  |  |  |
| 我的主治医生已经指导我应当如何戒烟  My doctor has advised me on how to stop smoking |  |  |  |
| 我的主治医生已经给我提供了饮食方面的建议  My doctor has advised me on what I should eat |  |  |  |
| 我的主治医生已经告诉我应该进行多大量的运动活动  My doctor has advised me on how much exercise I should do |  |  |  |
| 我的主治医生已经告诉我应该进行什么类型的运动  My doctor has advised me on what kind of exercise I should do |  |  |  |

**5、您认为以下内容对您患慢阻肺的影响有多大？How important do you think the following are in causing your lung condition?**

|  | 完全不重要 Not Important at All | 有点重要 Low Important | 重要 Important | 非常重要 Very Important | 我不知道  I don’t know |
| --- | --- | --- | --- | --- | --- |
| 冷/咳嗽/流感  Cold/Coughs/Flu |  |  |  |  |  |
| 吸烟My smoking |  |  |  |  |  |
| 吸二手烟  Second hand Smoke |  |  |  |  |  |
| 工作中的气体, 烟尘或灰尘  Gases, fumes or dust at work |  |  |  |  |  |
| 烹饪油烟Cooking Fumes |  |  |  |  |  |
| 生物燃料Biofuels |  |  |  |  |  |
| 空气污染Air pollution |  |  |  |  |  |
| 其他原因（请具体说明）  Other (Please specify) |  | | | | |

**6.您认为以下内容对您慢阻肺急性加重的影响有多大？（突然呼吸困难、咳嗽加重，痰/粘液颜色或量的变化）How important do you think the following are in causing your lung exacerbation?**

|  | 完全不重要 Not Important at All | 有点重要 Low Important | 重要 Important | 非常重要 Very Important | 我不知道  I don’t know |
| --- | --- | --- | --- | --- | --- |
| 冷/咳嗽/流感  Colds/Coughs/Flu |  |  |  |  |  |
| 吸烟My smoking |  |  |  |  |  |
| 吸二手烟  Second hand Smoke |  |  |  |  |  |
| 工作中的气体, 烟尘或灰尘  Gases, fumes or dust at work |  |  |  |  |  |
| 烹饪油烟Cooking Fumes |  |  |  |  |  |
| 空气污染Air pollution |  |  |  |  |  |
| 其他原因（请具体说明）  Other (Please specify) |  | | | | |

**7. 该问题关于您目前肺部疾病的管理。医生已经为您开具了以下哪种治疗方案？
This question is about the current management of your lung condition. Which of the following treatment options has been prescribed to you?**

|  | 是Yes |
| --- | --- |
| 日常吸入药 ⻓效支气管扩张剂(如噻托溴铵、沙美特罗) Daily Inhalers – Long acting bronchodilators (example: tiotropium, salmeterol) |  |
| 紧急吸入药短效支气管扩张剂(如沙丁胺醇、异丙托溴铵)  Emergency Inhalers - Short acting bronchodilators (example: salbutamol, ipratropium) |  |
| 口服药物/片剂 Oral Medication / Tablets |  |
| 抗生素 Antibiotics |  |
| 中药 Traditional Chinese Medicines |  |
| 监督下进行的运动项目 团体运动、骑自行车训练  Supervised Exercise Programme – Group exercise classes, Cycling training |  |
| 呼吸训练 缩唇呼吸、咳嗽训练、深呼吸  Breathing Exercises – pursed lip breathing, huff cough, deep breathing |  |
| 其他治疗手段（请详细说明）Other Treatments (Please Specify) | |
| 无 nothing | |

**8.以下运动你在空闲时间里一周平均可以做几次（每次超过15分钟）？During a typical 7-Day period (a week), how many times on average do you do the following kinds of exercise for more than 15 minutes during your free time? (write on each line the appropriate number)**

|  | Times per week |
| --- | --- |
| 1. 剧烈运动（心率加快）   （如跑步、慢跑、足球、壁球、篮球、越野滑雪、柔道、轮滑、快速游泳、快速长途骑自行车、剧烈强度乒乓球等）  STRENUOUS EXERCISE (HEART BEATS RAPIDLY) (e.g., running, jogging, , football, soccer, squash, basketball, cross country skiing, judo, roller skating, vigorous swimming, vigorous long distance bicycling, vigorous table tennis) |  |
| 1. 中等强度运动（不会疲惫）   （快走、棒球、网球、慢速骑自行车、排球、羽毛球、慢速游泳、高山滑雪、民间舞、广场舞、一般强度乒乓球、八段锦等）  MODERATE EXERCISE  (NOT EXHAUSTING) (e.g., fast walking, baseball, tennis, easy bicycling, volleyball, badminton, easy swimming, alpine skiing, popular and folk dancing, square dancing, easy table tennis, baduanjin ) |  |
| 1. 轻微运动（最小强度）   （瑜伽、射箭、岸边钓鱼、保龄球、掷马蹄铁、高尔夫、雪地摩托、慢走、太极等）  MILD/LIGHT EXERCISE (MINIMAL EFFORT) (e.g., yoga, archery, fishing from river bank, bowling, horseshoes, golf, snow-mobiling, easy walking, Tai Chi) |  |

**9. 这个问题涉及到目前肺部疾病管理情况。This question is about the current management of your lung condition.**

|  | 是Yes | 否No | 不确定或不适用 N/A |
| --- | --- | --- | --- |
| 我知道去何处治疗慢阻肺  I know where to go to get treatment for my lung condition |  |  |  |
| 我到医院很方便I can easily travel to the hospital |  |  |  |
| 我知道如何使用吸入药物I know how to use my inhalers |  |  |  |
| 我了解如何能正确地服用药物  I know how to take my medication correctly |  |  |  |
| 我了解当我的呼吸困难加重的时候应做什么  I know what to do when my breathing gets worse |  |  |  |

**10. 该问题主要关于您慢阻肺治疗的花销This question is about the cost of the treatment of your lung condition.**

|  | 不，我不能负担  No, I cannot afford it | 我可以负担一部分  I can afford some of it | 是，我可以负担全部  Yes, I can afford all of it |
| --- | --- | --- | --- |
| 我可以负担药品花销 |  |  |  |

**11. 我们正在考虑设计一项新的肺健康服务项目，该服务将包括慢阻肺患者的活动和健康教育计划，这些计划将在数周内定期举行。 它可以帮助慢阻肺患者减少呼吸困难，改善健康状态。 您有兴趣参加这类计划吗？ We are considering designing a new lung health service that will involve a programme of activity and education for people with lung conditions that will be delivered in regular sessions over a number of weeks. It could help reduce breathlessness and improve patient wellbeing. Would you be interested in taking part in this sort of programme?**

| 是的，当然愿意参加 Yes, definitely | 可能会愿意参加 Possibly | |  | 不愿意参加 No |
| --- | --- | --- | --- | --- |
| 如果不愿意，请给出原因If no, please give your reason |  |  | | |

**12. 您希望这些项目何时进行？（勾选所有符合条件的选项）When would you like the programme to take place? (Tick all that apply)**

| 周一-周五  Monday to Friday | 上午 - Morning |  |
| --- | --- | --- |
|  | 下午 – Afternoon |  |
|  | 晚上 – Evening |  |
| 周末 Weekends | 上午 - Morning |  |
|  | 下午 – Afternoon |  |
|  | 晚上 – Evening |  |

**13. 这些项目通常至少包括12次课程，每周2次，为期6-8周。 您愿意参加多少次？These programmes usually involve at least 12 sessions - 2 sessions a week for 6-8 weeks. How many sessions would you be happy to attend?**

| 每周1次，进行8至12周 1 session a week for 8-12 weeks |  |
| --- | --- |
| 每周2次，进行6至8周 2 sessions a week for 6-8 weeks |  |
| 每周1次，进行6至8周 1 session a week for 6-8 weeks |  |
| 每周2次，6周以下 2 sessions a week for less than 6 weeks |  |

**14. 您希望如何安排项目小组的活动？How would you like the programme groups to be organised?**

| 我在小组内开始项目，并且我们每周参加相同的课程  I start the programme in a group and we attend the same sessions each week |  |
| --- | --- |
| 我方便时会参与项目，但每周只参加相同课程 I start the programme when it is convenient for me but attend the same session each week |  |
| 我每周参加最方便的课程 I attend the most convenient session each week |  |
| 我自行完成这些项目 I complete the programme by myself |  |

**15. 您希望这些项目在何处进行？Where would you like the programme to take place?**

| 大型医院 Main Hospital |  |
| --- | --- |
| 社区医院Community Hospital |  |
| 我的家My Home |  |
| 其他（请详细描述）Other (please specify) | |

**16. 如果您参与肺健康服务，请选出您最想学习的5个内容。Please tick the five things you would most like to take part in if you were to sign up to a lung health service**

| 呼吸技术：缩唇呼吸、咳嗽训练、深呼吸、腹式呼吸Breathing techniques - pursed lip breathing, huff cough, deep breathing,abdominal breathing |  |
| --- | --- |
| 督导下的有氧运动：跑步，骑自行车等Supervised Aerobic Exercise (Running, cycling) |  |
| 监督下的负重运动：举臂、二头肌功能锻炼、弓步Supervised Exercises with weights – Arm raises, Bicep curls, Lunges |  |
| 指导下的放松性练习：太极拳，气功等Guided Relaxation Exercise (Tai Chi, Qi Gong ) |  |
| 关于我应当做什么运动的大体的建议General advice about what exercise I should do |  |
| 关于饮食的建议Advice about what I should eat |  |
| 关于戒烟的建议Advice about stopping smoking |  |
| 关于慢阻肺的健康教育Education about my lung condition |  |
| 吸入药物使用方法Inhaler Technique Advice |  |
| 自我管理技术Self-Management Techniques |  |
| 关于在突然发生呼吸困难时我应当做什么的建议Advice about what to do if I become suddenly breathless |  |
| 关于焦虑和抑郁症的咨询Counselling for anxiety and depression |  |
| 关于如何应对工作和慢阻肺的建议Advice about coping with work and my lung condition |  |
| 对我的家庭和护理的支持和建议Support and advice for my family and carers |  |
| 其他：___________others |  |

**17.当你参加运动项目时，您希望在医生/护士监督指导下进行吗？Would you prefer to carry out the exercise, part of the program, with or without supervision?**

| 希望监督指导With supervision |  |
| --- | --- |
| 不希望监督指导 Without supervision |  |
| 都可以 both is ok |  |

**18.请选出决定您是否参与肺健康服务的3个最重要的因素
Please tick the three most important factors that would influence your decision to sign up to a lung health service**

| 我是否需要付费 If I had to pay for the sessions |  |
| --- | --- |
| 距离家里的距离 How far away I live |  |
| 交通是否便利 How easy it is to travel to the sessions |  |
| 参与到这些项目后我是否会耽误工作或其他重要活动  If the sessions meant I missed work or other important activities. |  |
| 项目持续多少周 How many weeks the programme lasts |  |
| 每周有多少次课程How many sessions per a week |  |
| 包括哪些运动或活动的类型The type of exercise or activity involved |  |
| 我的主治医生是否推荐我参加这项活动If my doctor recommended the programme |  |
| 我认识的其他人是否参与活动 If someone I knew was also participating |  |

**19.如果你参加肺健康服务，你每次愿意支付多少费用？How much are you willing to pay for the lung health service?**

| 费用/次 cost/one time |  |
| --- | --- |
| <10元 <10RMB |  |
| 10-50 元10-50 RMB |  |
| 50-100元50-100 RMB |  |
| 100-200元100-200RMB |  |
| 其他金额 others |  |

感谢您完成了这份问卷

我们感谢您花费时间来回答我们的问题

请将问卷交还给我们研究小组的工作人员

Thank you for completing the questionnaire!

We appreciate that you have taken the time to answer our questions.

Please return the questionnaire to a member of our research team

|  | **Part 1** | | | **Part 2** | | | | | | | | | | | | | | | | | | | |
| --- | --- | --- | --- | --- | --- | --- | --- | --- | --- | --- | --- | --- | --- | --- | --- | --- | --- | --- | --- | --- | --- | --- | --- |
| **Question** | **9** | **10** | **13** | **1**^b^ | **2**^a,c^ | **3** | **4** | **5** | **6** | **7** | **8**^a^ | **9**^d^ | **10** | **11** | **12** | **13** | **14** | **15** | **16** | **17**^a^ | **18** | **19**^a^ |  |
| Beijing, n | 3 |  | 3 | 1 |  | 1 | 2 | 4 | 3 | 1 |  | 2 | 1 | 1 | 18 | 18 | 18 | 18 | 18 |  | 20 |  |  |
| Chengdu, n |  |  |  |  |  |  | 1 |  |  |  | 2 | 1 | 1 |  | 2 | 1 |  |  | 2 | 2 | 1 |  |  |
| Guangzhou, n | 2 | 1 |  |  |  |  | 1 | 1 | 3 |  |  |  |  |  | 3 | 3 | 4 | 3 | 4 | 2 | 2 | 3 |  |
| Shenyang, n |  |  |  | 3 | 5 | 1 | 1 |  |  |  |  | 7 |  |  | 2 | 1 | 1 | 1 | 2 | 2 | 3 | 2 |  |
| **Overall, n** | **5** | **1** | **3** | **4** | **5** | **2** | **5** | **5** | **6** | **1** | **2** | **10** | **2** | **1** | **25** | **23** | **23** | **22** | **26** | **6** | **26** | **5** |  |
| % | 2.0 | 0.4 | 1.2 | 1.6 | 3.2 | 0.8 | 2.0 | 2.0 | 2.4 | 0.4 | 1.3 | 4.0 | 0.8 | 0.4 | 10.0 | 9.2 | 9.2 | 8.8 | 10.4 | 3.9 | 10.4 | 3.2 |  |
| a: New question added for Chengdu, Guangzhou & Shenyang (n=155)  Data were imputed following discussions with local researchers revealed that:  b: 2 participants did not think they had COPD so did not answer this Y/N question “I know the name of my lung disease”. Their responses were imputed as “No”.  c: 4 participants who left this question blank (When were you diagnosed with COPD) were diagnosed with COPD during the Breathe Well Programme^^[[2]](#footnote-2)^^  in 2019 so their responses were imputed as “In the last year”.  d: In response to the list of statements, 4 participants only ticked “Yes” for what they knew, remaining items that they did not know were left blank. Blank items were imputed as “No”. | | | | | | | | | | | | | | | | | | | | | | | |

Supplementary Table 5 Missing data (N=251)

**Topic guide - Patients**

**Before focus groups begin:**

- Welcome and greet participants
- Give out refreshments
- Distribute consent forms and info sheets / answer general queries

**Introductions (5-10 minutes)**

Hello everyone and thank you for agreeing to take part in this group discussion today.

My name is Hui and I am a master’s student from General Medicine Department of Peking University First Hospital. We are here today to find out about your thoughts and opinions on your lung condition.

We want to find out how your lung condition affects your life now and also want to ask your opinion about lung health service that we want to design in the future.

We will be recording today’s discussion and this recording will be used to make a written transcript.

Everything said in this room will remain confidential and you will not be identified in the transcript so please express yourself freely.

The discussion will take up to 2.5 hours. We will have a break after an hour for refreshments.

After the group discussion, there will be an educational session. If you have any question, there will be an opportunity to ask questions about your lung condition.

Does anyone have any questions?

Ground Rules

“To allow our conversation to flow more freely, I’d like to go over some ground rules.

1. Only one person speaks at a time. This is very important as our goal is to make a written transcript of our conversation today and it is difficult to do this when two people speak at once.
2. Everyone doesn’t have to answer every single question, but we’d like to hear from each of you today as the discussion progresses.
3. This is a confidential discussion in that we will not report your names or who said what. Names of participants will not even be included in the final report about this meeting. It also means, except for the report that will be written, what is said in this room stays in this room.
4. This is a group discussion so feel free to reply to each other’s comments. You do not need permission to speak so please feel free to speak openly.
5. There are no “wrong answers,” just different opinions. Say what is true for you, even if you’re the only one who feels that way. Don’t let the group sway you. But if you do change your mind, let me know.
6. Let me know if you need a break. The bathrooms are [location]. Feel free to enjoy a beverage and a snack.

**Tell us your name and where you are from**

**Section 1 (25-30 minutes)**

1. **When were you diagnosed with COPD**

**Describe COPD to me in your own words (What kind of disease is COPD?)**

**2. Can you tell the group about the medical care you receive for your COPD**

**Section 2**

We are considering designing a new lung health service that will involve a programme of activity and education for people with lung conditions that will be delivered in regular sessions over a number of weeks. In the UK these programmes are becoming increasingly popular as they can help to reduce breathlessness and improve patient wellbeing. You would be referred to this service when you are first diagnosed with COPD and would receive different levels of support as your disease progresses.

- **What are your first impressions of such a service?**

**Elements of a lung health service (45-60 minutes)**

*Show pictures of smoking, education, physical activity, planning/coping strategies and well-being

1. We have 5 different elements of a lung health service here:
   - **Which one do you think is most important?**
   - **Why do you think this is important?**

Further Discussion Points

**Education: What sort of information would you like to be included in the education sessions?**

***Show picture examples of different types of exercise – cycling, circuits, tai chi?, walking**

**Physical Activity: How do you feel about this type of physical activity? What sort of activities would you like to take part in?**

- - What kind of exercise? Cycling, Running, Dance, Tai Chi/Qi Gong, Walking/Hiking
  - Where? Indoors, Outdoors, Gym, Community Centre, At Home
    - How far would you be willing to travel?
  - How would you feel about having a homework element?
    - Homework diary to record what exercises they do at home

**Self Management: What self-management techniques would help you to manage your COPD on a daily basis?**

**Smoking: Do you think it is important for patients with COPD to stop smoking?**

- Prompts
  - What information helps people to stop smoking?
  - If you were offered medications to quit smoking, would you want to take it?

**Well-being: In England, we find that lots of patients with long term health conditions experience low mood and feel anxious. What do you think about this?**

**Design of the service (30-45 minutes)**

**1. What would encourage someone to use a lung health service?**

- Prompts
  - The name of the programme
  - Programme length and frequency
  - Location of programme
    - Outdoors - weather
  - Who else was taking part?
  - Time of the year? - holidays, Chinese New Year

1. **What would discourage someone from using this kind of service?**
   - Cost of the programme?
   - Programme length and frequency
   - If their breathing was better or worse than usual?
2. **In the UK, patients usually visit this kind of service on a regular basis. How often would you be willing to attend?**
3. **Do you think that family members or carers should be involved in the sessions?**
   - What support do you think your family needs, if any?
4. **A lung health service could provide lots of different activities and support. We would now like you to pick your five most important elements from the list in front of you. Please take your time:**
   - Breathing techniques
   - Supervised Aerobic Exercise (Running, cycling etc)
   - Supervised Exercises with weights
   - Guided Relaxation Exercise (Tai Chi, Qi Gong etc)
   - General advice about what exercise I should do
   - Advice about what I should eat
   - Advice about stopping smoking
   - Education about my lung condition
   - Inhaler Technique Advice
   - Self-Management Techniques
   - Advice about what to do if I become suddenly breathless
   - Counselling for anxiety and depression
   - Advice about coping with work and my lung condition
   - Support and advice for my family and carers

**Why do you think these elements are most important?**

**Thank you for sharing all your thoughts and experiences with us today. It has been very interesting. I have asked all my questions but is there anything else you would like to discuss?**

**Thank participants**

**Give out gifts and collect in materials**

**Topic guide - GPs**

**Before focus groups begin:**

- Welcome and greet participants
- Give out refreshments
- Distribute consent forms and info sheets / answer general queries

**Introductions (5-10 minutes)**

Hello everyone and thank you for agreeing to take part in this group discussion today.

My name is Hui and I am a master’s student from General Medicine Department of Peking University First Hospital. We are here today to find out about the current management of COPD in China and also discuss with you a future lung health service we would like to design in the future.

We have invited you all here because you have experience with managing patients with COPD and we are interested to hear your thoughts.

We will be recording today’s discussion and this recording will be used to make a written transcript.

Everything said in this room will remain confidential and you will not be identified in the transcript so please express yourself freely.

The discussion will take up to 2.5 hours. We will have a break after an hour for refreshments.

After the group discussion, there will be a presentation about pulmonary rehabilitation and self-management services we use in the UK and there will be an opportunity to ask any questions.

Does anyone have any questions?

“To allow our conversation to flow more freely, I’d like to go over some ground rules.

1. Only one person speaks at a time. This is doubly important as our goal is to make an written transcript of our conversation today. It is difficult to capture everyone’s experience and perspective on our audio recording if there are multiple voices at once
2. Please avoid side conversations.
3. Everyone doesn’t have to answer every single question, but I’d like to hear from each of you today as the discussion progresses.
4. This is a confidential discussion in that I will not report your names or who said what to your colleagues or supervisors. Names of participants will not even be included in the final report about this meeting. It also means, except for the report that will be written, what is said in this room stays in this room.
5. We stress confidentiality because we want an open discussion. We want all of you to feel free to comment on each other’s remarks without fear your comments will be repeated later and possibly taken out of context.
6. There are no “wrong answers,” just different opinions. Say what is true for you, even if you’re the only one who feels that way. Don’t let the group sway you. But if you do change your mind, let me know.
7. Let me know if you need a break. The bathrooms are [location]. Feel free to enjoy a beverage and a snack

**Section 1 (25-30 minutes)**

1. **Can you each introduce yourself to the group, and tell us where you work and what experience you have with managing COPD patients?**

- How many patients do you manage with COPD?
- What is your role in their medical care?

1. **What are your thoughts about the current management of COPD in your workplace?**

**Section 2**

In some countries, pulmonary rehabilitation and self-management programmes involving exercise and education are used to help improve symptoms and patient well-being. These services are usually based in the community and patients attend a regular group session each week. This service can provide different levels of support depending on the severity of the patients’s COPD. We want to know your thoughts on the design and feasibility of a community “lung health” programme which would incorporate aspects of self-management and pulmonary rehabilitation.

**Elements of a lung health service (45-60 minutes)***

1. **Programmes like this usually include an educational element, so a topic would be discussed each week for about 30 minutes. In your experience, what information do people with COPD need to know? What do you think patients with COPD should be taught about their condition?**
2. **These services usually involve some element of exercise or activity. In the UK, patients attend group exercise classes. *Show video/pictures**

**What do you think of this? What sort of activities do you think patients would be willing to take part in?**

- Prompts
  - What kind of exercise? Cycling, Running, Dance, Tai Chi/Qi Gong, Walking/Hiking
  - Where? Indoors, Outdoors, Gym, Community Centre, At Home
  - Homework?
    - Homework diary to record what exercises they do at home

1. **Let’s talk about self-management now. Patients who are able to manage their own health often have the best outcomes. What support do patients need so they can manage their own lung condition?**
2. **Smoking is a major cause of COPD. In your opinion, how can we help patients to stop smoking?**
3. **In the UK, patients with long term health problems often feel anxious about their health and experience low mood. How could this service help patients with anxiety or depression?**

- Prompts
  - Signposting to another service
  - Psychological intervention
    - Individual
    - Group sessions
  - Would the GPs feel comfortable delivering this intervention?

**Design of the service (30-45 minutes)**

**The idea behind this service is that you would be able to refer your patients to the lung health centre when they are first diagnosed. They would receive different levels of support depending on the severity of their disease and the support provided would change as their disease progressed. Patients who are newly diagnosed could receive education and support with smoking cessation; patients with more severe symptoms could attend exercise sessions and self-management classes.**

**6. Patients would need to regularly attend exercise and education classes. How often do you think patients would be willing to attend these classes?**

- Prompt
  - Usually 12 sessions over 6-8 weeks - more or less?
  - Twice weekly
  - Morning, Afternoon, Evening, Weekend?

**7. Who do you think would be best to give these classes?**

**8. Do you think the group dynamic would make a difference to whether patients wanted to attend?**

- Prompts
  - Mixed sex group?
  - Working age patients separated from retired patients?
  - Should family and carers attend?

**9. What would be difficult about setting up a lung health service in the community?**

- Prompts
  - Patient engagement - will they want to sign up?
  - Cost of the programme - co-payment?

**10. Thank you for sharing all your thoughts and experiences with us today. It has been very interesting. I have asked all my questions but is there anything else you would like to discuss?**

**Thank participants**

**Give out small gifts and collect in materials**

1. **Tertiary hospitals** have the comprehensive capabilities of medical, teaching and scientific research, which aim to provide specialist medical services and solve critical and difficult diseases for patients across the country [↑](#footnote-ref-1)
2. Pan, Z.*, et al.* Study to evaluate the effectiveness and cost-effectiveness of different screening strategies for identifying undiagnosed COPD among residents (≥40 years) in four cities in China: protocol for a multicentre cross-sectional study on behalf of the Breathe Well group. *BMJ Open* **10**, e035738 (2020). [↑](#footnote-ref-2)
