# Supplementary material for: Community lung health service design for COPD patients in China by the Breathe Well group
Source: NPJ Prim Care Respir Med. 2022 Aug 19;32:27. doi: 10.1038/s41533-022-00286-8 (PMC9388970; doi:10.1038/s41533-022-00286-8)
Supplement: Supplementary file 2 — REPORTING SUMMARY [file 41533_2022_286_MOESM2_ESM.pdf]

nature portfolio

Corresponding author(s): Dr Chunhua

Last updated by author(s): Jan 24, 2022

Reporting Summary

Nature Portfolio wishes to improve the reproducibility of the work that we publish. This form provides structure for consistency and transparency in reporting. For further information on Nature Portfolio policies, see our Editorial Policies and the Editorial Policy Checklist.

Statistics

For all statistical analyses, confirm that the following items are present in the figure legend, table legend, main text, or Methods section.

n/a	Confirmed	
☐	☒	The exact sample size (n) for each experimental group/condition, given as a discrete number and unit of measurement
☒	☐	A statement on whether measurements were taken from distinct samples or whether the same sample was measured repeatedly
☒	☐	The statistical test(s) used AND whether they are one- or two-sided<br>*Only common tests should be described solely by name; describe more complex techniques in the Methods section.*
☒	☐	A description of all covariates tested
☒	☐	A description of any assumptions or corrections, such as tests of normality and adjustment for multiple comparisons
☒	☐	A full description of the statistical parameters including central tendency (e.g. means) or other basic estimates (e.g. regression coefficient) AND variation (e.g. standard deviation) or associated estimates of uncertainty (e.g. confidence intervals)
☒	☐	For null hypothesis testing, the test statistic (e.g. F, t, r) with confidence intervals, effect sizes, degrees of freedom and P value noted<br>*Give P values as exact values whenever suitable.*
☒	☐	For Bayesian analysis, information on the choice of priors and Markov chain Monte Carlo settings
☒	☐	For hierarchical and complex designs, identification of the appropriate level for tests and full reporting of outcomes
☒	☐	Estimates of effect sizes (e.g. Cohen's d, Pearson's r), indicating how they were calculated

Our web collection on statistics for biologists contains articles on many of the points above.

Software and code

Policy information about availability of computer code

Data collection	Quantitative data were entered from paper to REDCap, Chengdu FGs were conducted via Tencent Conference 2.0
Data analysis	STATA v 15.1 for quantitative data

For manuscripts utilizing custom algorithms or software that are central to the research but not yet described in published literature, software must be made available to editors and reviewers. We strongly encourage code deposition in a community repository (e.g. GitHub). See the Nature Portfolio guidelines for submitting code & software for further information.

Data

Policy information about availability of data

All manuscripts must include a data availability statement. This statement should provide the following information, where applicable:

- Accession codes, unique identifiers, or web links for publicly available datasets
- A description of any restrictions on data availability
- For clinical datasets or third party data, please ensure that the statement adheres to our policy

The data supporting the findings of this study are available from the corresponding author upon written application and completion of appropriate data sharing agreements.

Field-specific reporting

Please select the one below that is the best fit for your research. If you are not sure, read the appropriate sections before making your selection.

☐ Life sciences ☒ Behavioural & social sciences ☐ Ecological, evolutionary & environmental sciences

For a reference copy of the document with all sections, see nature.com/documents/nr-reporting-summary-flat.pdf

Behavioural & social sciences study design

All studies must disclose on these points even when the disclosure is negative.

Study description	Mixed-method study
Research sample	Patients from urban community healthcare centres in Beijing, Chengdu, Guangzhou and Shenyang, China with a spirometry-diagnosed COPD, and GPs with experience of managing COPD were eligible. We collect a representative sample of 251 survey respondents, who had a mean age of 67.9 (SD 9.1) years, 183 (72.9%) were male. 39 patients (27 male) and 30 GPs (6 male) participated in ten separate focus groups (FGs). FG patients had a mean age of 66.3 (SD 7.8) years. FG GPs had a mean age of 39.5 (SD 8.0) years.
Sampling strategy	In order to maximise diversity within the qualitative data we purposively sampled different kinds of people and analysed the data alongside collection to ensure data saturation, that is, the point at which no new themes were emerging. No formal sample size calculation was undertaken for quantitative data but a pragmatic approach was taken to collect a representative sample of 100 patients in Beijing and 50 patients in each of the other three cities, around 250 in total.
Data collection	Pen and paper, audio equipment. Nobody was present besides the participants and the researcher. Researcher was blind to hypothesis during data collection.
Timing	February 2019 to June 2020
Data exclusions	Delete 1 patient (ID:0107) because the patient had Asthma instead of COPD. Exclusion criteria were pre-establised.
Non-participation	Of 278 eligible COPD patients identified and invited, 23 declined to attend CHCs because of COVID-19 (Figure 1). Of the 255 patients who provided consent, 4 withdrew because they did not have enough time to complete the questionnaire.
Randomization	Participants were not allocated into experimental groups.

Reporting for specific materials, systems and methods

We require information from authors about some types of materials, experimental systems and methods used in many studies. Here, indicate whether each material, system or method listed is relevant to your study. If you are not sure if a list item applies to your research, read the appropriate section before selecting a response.

Materials & experimental systems

n/a	Involved in the study	
☒	☐	Antibodies
☒	☐	Eukaryotic cell lines
☒	☐	Palaeontology and archaeology
☒	☐	Animals and other organisms
☐	☒	Human research participants
☒	☐	Clinical data
☒	☐	Dual use research of concern

Methods

n/a	Involved in the study	
☒	☐	ChIP-seq
☒	☐	Flow cytometry
☒	☐	MRI-based neuroimaging

Human research participants

Policy information about studies involving human research participants

Population characteristics	See above
Recruitment	Eligible patients were identified through hospital attendance records and through local GP lists. Researchers then telephoned potential participants to invite them to participate.
Ethics oversight	Ethics Review Board at Peking University First Hospital [No: 2019/52, 13/03/2019] and the Internal Review Ethics Committee at University of Birmingham [IREC2018/1413420, 11/11/2019].

Note that full information on the approval of the study protocol must also be provided in the manuscript.
